# Quorum sensing and antibiotic resistance in polymicrobial infections

**DOI:** 10.1080/19420889.2024.2415598

**Published:** 2024-10-17

**Authors:** Sunny Cui, Esther Kim

**Affiliations:** a Department of Biological Sciences, Dartmouth College, Hanover, NH, USA; b Arts and Science, University of Toronto, Toronto, ON, Canada

**Keywords:** Bacteria communication, biofilm formation, biofilms, biological communication, polymicrobial infections, quorum inhibitors, quorum quenchers, quorum sensing, quorum sensing inhibitors, signal molecules

## Abstract

Quorum sensing (QS) is a critical bacterial communication system regulating behaviors like biofilm formation, virulence, and antibiotic resistance. This review highlights QS’s role in polymicrobial infections, where bacterial species interactions enhance antibiotic resistance. We examine QS mechanisms, such as acyl-homoserine lactones (AHLs) in Gram-negative bacteria and autoinducing peptides (AIPs) in Gram-positive bacteria, and their impact on biofilm-associated antibiotic resistance. The challenges uniquely associated with polymicrobial infections, such as those found in cystic fibrosis lung infections, chronic wound infections, and medical device infections, are also summarized. Furthermore, we explore various laboratory models, including flow cells and dual-species culture models, used to study QS interactions in polymicrobial environments. The review also discusses promising quorum sensing inhibitors (QSIs), such as furanones and AHL analogs, which have demonstrated efficacy in reducing biofilm formation and virulence in laboratory and clinical studies. By addressing the interplay between QS and antibiotic resistance, this paper aims to advance therapeutic strategies that disrupt bacterial communication and improve antibiotic efficacy, ultimately mitigating the global challenge of antibiotic resistance in polymicrobial infections.

## Introduction

**Figure 1.**
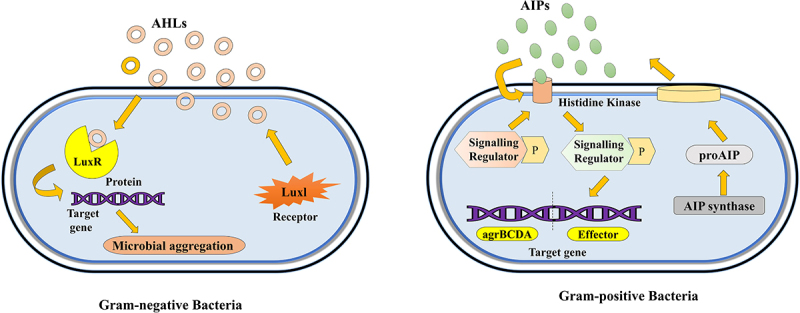
Quorum sensing via AHLs in gram-negative and AIPs in gram-positive bacteria.

Quorum sensing (QS) is a sophisticated cell-to-cell communication mechanism employed by bacteria to synchronize behavior in response to population density [1]. This process entails the synthesis, release, and detection of signaling molecules. Upon reaching a critical threshold concentration, a coordinated cell response is often triggered that governs various collective behaviors within the bacterial community, including regulating biofilm formation, virulence factor production, gene expression, bioluminescence, among others [2,3]. QS was first discovered in marine bacteria *Vibrio fischeri* in the 1960s [4]. Since then, QS research has since extended to numerous bacterial species, encompassing both Gram-positive and Gram-negative bacteria. In Gram-negative bacteria, QS typically involves acyl-homoserine lactone (AHL) molecules, while Gram-positive bacteria predominantly utilize autoinducing peptides (AIPs) [5–7] (Figure 1). Despite the diversity in QS signaling molecules, the fundamental principle of QS remains consistent, enabling bacteria to detect their population density and modulate behavior to optimize survival strategies under fluctuating environmental conditions.

Biofilm formation, a critical QS-regulated collective behavior, involves the development of structured bacterial communities encased in a self-produced extracellular matrix adhering to surfaces, providing a protective environment for the bacteria [8]. Within biofilms, bacteria exhibit increased resistance to environmental stresses, including antibiotic treatments [9,10]. Modern research has thus focused on QS’s role in biofilm development, particularly in regulating the synthesis of extracellular polymeric substances (EPS) and enzymes. Understanding and combating these factors are essential for reducing biofilm persistence in hostile environments, making infection treatment more effective. In the modern day, antibiotic resistance from biofilms remain a major global public health challenge. The World Health Organization (WHO) has declared antibiotic resistance a global health emergency, underscoring the need for urgent and coordinated actions to mitigate such threats [11]. The economic burden on healthcare systems is also substantial. QS-induced biofilm antibiotic resistance frequently necessitates more expensive and prolonged therapies, additional diagnostic testing, and extended hospital stays [12–14]. Moreover, antibiotic resistance can lead to treatment failures, requiring the use of last-resort antibiotics [15,16]. These last-resort antibiotics have been shown to have more severe side effects and lower efficacy [17,18]. Finally, biofilm-induced antibiotic resistance poses an economic burden beyond healthcare, affecting agricultural productivity and food security [19,20].

QS plays a pivotal role in the development of antibiotic resistance in polymicrobial infections involving multiple microbial species [21,22]. In such infections, QS mediates interactions between different bacterial species, enhancing their collective ability to resist antibiotic treatments. Given the multifaceted interplay between QS and antibiotic resistance in polymicrobial infections, understanding this connection is crucial for developing innovative therapeutic strategies. Targeting QS pathways with quorum sensing inhibitors (QSIs) could potentially render bacteria more susceptible to antibiotic treatments. This review aims to elucidate the complex relationship between QS and antibiotic resistance and highlight recent advancements in therapeutic interventions targeting QS systems in polymicrobial infections.

## Mechanisms of quorum sensing

QS signals are diverse and vary among bacterial species, with the three main types being acyl-homoserine lactones (AHLs) in Gram-negative bacteria, oligopeptides in Gram-positive bacteria, and autoinducer-2 (AI-2), which can serve as a universal signaling molecule across different bacterial species [5,23–25]. Other more recently discovered QS signaling molecules include Alkyl Quinolones (PQS), Diffusible Signal Factors (DSF), Autoinducer-3 (AI-3), and Cholera Autoinducer-1 (CAI-1).

Acyl-homoserine lactones, or AHLs, are commonly synthesized by LuxI-family proteins, though other synthases, such as LuxM and AinS, also produce AHLs. They serve as the primary QS signals in certain Gram-negative bacteria. For instance, the LuxI/LuxR system in *V. fischeri* involves the synthesis of AHLs by the LuxI protein, which diffuse out of the cell, accumulate in the environment, and reenter the cell to bind LuxR receptors, activating gene transcription leading to bioluminescence [26,27]. Similarly, in *Pseudomonas aeruginosa*, QS involves two primary systems: LasI/LasR and RhlI/RhlR [28,29]. The LasI/LasR system uses *N*-(3-oxododecanoyl)-homoserine lactone (3-oxo-C12-HSL) as the signaling molecule, while the RhlI/RhlR system uses N-butanoyl-homoserine lactone (C4-HSL). The LasI/LasR system hierarchically activates the RhlI/RhlR system, with LasI synthesizing 3-oxo-C12-HSL and binding to the LasR receptor, activating genes involved in production of elastase and other proteases that degrade host tissues and immune defenses, enhancing bacterial invasion. This hierarchical QS network allows *P. aeruginosa* to fine-tune its response to environmental conditions and enhance pathogenicity. Furthermore, *P. aeruginosa* can integrate other QS signaling molecules such as alkyl quinolones with its AHL systems [30,31]. PQS is regulated by the LasI/LasR system, which controls the *pqsABCDE* operon responsible for PQS synthesis. Once produced, PQS binds to the PqsR receptor in the cytoplasm, activating genes involved in iron chelation and neutralization of reactive oxygen species. PQS also influences the RhlI/RhlR system by modulating C4-HSL production, thereby integrating with the AHL systems to fine-tune *P. aeruginosa*’s pathogenic response and enhance its ability to thrive under stressful conditions [32,33] (Figure 3).

Meanwhile, in other Gram-negative bacteria such as *Vibrio cholerae* and *Vibrio campbellii* (formerly *Vibrio harveyi*) a more complex QS system is present, integrating several signaling pathways. Rather than solely relying on AHL-based signaling, these *Vibrio* species can include other signaling molecules such as AI-2 and CAI-1 [34,35]. AI-2 is detected by the LuxPQ system, while CAI-1 is sensed by the CqsS receptor. In *V. cholerae*, quorum sensing pathways are controlled by transcription of the master regulator HapR, which is activated at high cell density to suppress virulence gene expression and promote biofilm dispersal [34]. In *V. campbellii*, the QS system employs a LuxM/LuxN pathway, where LuxM synthesizes 3-hydroxy-C4-HSL, which is detected by the LuxN receptor, along with integration of AI-2 and CAI-1 signaling pathways [35]. These complex signaling cascades allow response to diverse environmental conditions, fine-tuning behaviors such as bioluminescence, virulence, and biofilm formation.

In Gram-positive bacteria, QS signals are typically oligopeptides synthesized as precursor proteins and processed into active forms by peptidases [24]. These peptides are secreted into the environment and detected by membrane-bound histidine kinase receptors, which undergo autophosphorylation upon binding the oligopeptide initiating changes in gene expression through response regulators. An example of such system using this kind of signaling is the Agr system in Gram-positive *Staphylococcus aureus*, encoded by the *agrBDCA* operon [36,37]. The AgrD precursor is processed by AgrB to produce the active autoinducing peptide (AIP). AIP is then secreted and binds to the AgrC receptor, a membrane-bound histidine kinase, triggering its autophosphorylation. The phosphorylated AgrC transfers the phosphate group to the response regulator AgrA, which in turn binds to the P3 promotor of the *RNAIII* operon. The RNAIII transcript modulates the expression of numerous target genes, including those involved in toxin production and other virulence factors [38,39]. Another instance of a similar mechanism is the ComX/ComP/ComA QS system in Gram-positive *Bacillus subtilis*, controlling competence development and sporulation [40–42]. In this system, the signaling peptide ComX is detected by the ComP histidine kinase receptor, leading to autophosphorylation and subsequent phosphorylation of the ComA response regulator, which then activates genes involved in DNA uptake and incorporation. This process can facilitate horizontal gene transfer (HGT), enhancing the spread of antibiotic resistance genes among bacterial populations. In polymicrobial infections, QS-mediated HGT can lead to the dissemination of resistance genes across different species, complicating treatment strategies that revolve around antibiotic specificity [43].

There also exists several quorum sensing molecules used by both Gram-negative and Gram-positive bacteria. AI-2 is a universal signaling molecule synthesized by the LuxS enzyme, used by both Gram-positive (*Streptococcus mutans*, *B. subtilis*, *S. aureus*, etc) and Gram-negative (*Escherichia coli*, *V. cholerae, Salmonella enterica*) species [25,44,45]. The biosynthesis of AI-2 involves the conversion of S-adenosylmethionine to 4,5-dihydroxy-2,3-pentanedione (DPD), which then cyclizes to form the active signaling molecule [46]. In Gram-negative bacteria, such as *V. campbellii* BB120, the AI-2 molecule is detected by the LuxPQ receptor system [47–49]. In Gram-negative *E. coli*, the Lsr system is used to sense and respond to AI-2, allowing for intricate interactions in polymicrobial environments [50,51]. In Gram-positive bacteria, like *B. subtilis*, AI-2 can indirectly influence competence and sporulation by interacting with the aforementioned ComQXPA systems [52,53]. Similarly, AI-2 produced in the LuxS pathway of Gram-positive *S. mutans* regulates acid tolerance, which aid in its survival in the oral cavity [54]. Due to AI-2’s ability to serve as a signaling molecule in both Gram-negative and Gram-positive bacteria, it is heavily implicated in interspecies communication. For instance, in mixed bacterial communities of *E. coli* and *S. enterica*, *E. coli* can sense AI-2 produced by *S. enterica*, and vice versa, leading to changes in gene expression and competitive interactions [55,56].

QS signaling molecules are not just limited to communication between bacteria species. Certain autoinducers such as autoinducer-3 (AI-3) are more closely involved in interkingdom signaling, serving as a communication molecule between bacteria and non-bacteria host organisms [57]. AI-3 is detected by two-component systems such as QseC/QseB, where QseC is the sensor kinase and QseB is the response regulator [58,59]. Upon binding AI-3, QseC autophosphorylates and transfers the phosphate to QseB, which then regulates genes involved in motility and virulence

One of the more recent signaling molecules to be discovered include a group of fatty acid-based signaling molecules known as Diffusible Signal Factor (DSF) signals [60–62]. They are emerging as important mediators of interspecies communication and have been studied in species such as Xanthomonas campestris. DSF molecules are cis-2-unsaturated fatty acids synthesized by the RpfF enzyme and detected by the RpfC/RpfG two-component system. Other notable fatty acid-based signals include cis-2-decenoic acid in *P. aeruginosa* and A-factor in *Streptomyces*.

## Quorum sensing and antibiotic resistance

The interplay between quorum sensing (QS) and antibiotic resistance is complex. A thorough understanding of these mechanisms will be critical for developing strategies to combat antibiotic-resistant infections, elucidating how bacteria protect themselves, enhance resistance through interspecies communication, and facilitate the spread of resistance genes.

One of the main ways quorum sensing (QS) promotes antibiotic resistance is by influencing biofilm formation, primarily through the production of extracellular polymeric substances (EPS) and factors like polysaccharides and exopolysaccharides. This leads to a self-produced extracellular matrix that encases bacterial communities, protecting against antibiotics and the host immune system by reducing their diffusion rates [1,3,22]. Within these biofilm communities, the gradients of nutrients, oxygen, and waste create microenvironments where cells can enter a slow-growing or dormant state, making them less susceptible to antibiotics. Among these, persister cells – dormant variants within the biofilm – exhibit transient antibiotic tolerance, allowing them to survive treatment and repopulate the biofilm once the antibiotic pressure is removed [63]. This persistence of dormant, antibiotic-tolerant cells is a major contributor to recurrent infections. QS signaling is implicated in persister cell formation, with certain QS-regulated genes such as toxins-antitoxins modules (TA systems) and ppGpp synthesis genes playing key roles [64,65]. TA systems can induce dormancy and stress response pathways, such as such as the HipBA and MazEF systems. Meanwhile, the synthesis of the alarmone ppGpp under QS regulation, primarily through the RelA and SpoT enzymes, can help bacterial adaptation by downregulating ribosomal RNA synthesis and reducing metabolic activity to conserve energy and enhance dormancy.

The composition of the EPS matrix can dictate antibiotic resistance profiles. The polysaccharide Psl in *P. aeruginosa* increases resistance to colistin, polymyxin B, tobramycin, and ciprofloxacin by forming a barrier that reduces antibiotic penetration via electrostatic interactions. This effect also extends to non-Psl producers when co-cultured with *P. aeruginosa*, like *E. coli* and *S. aureus*, enhancing their resistance within biofilms [66]. Polymicrobial biofilms are also often more antibiotic resistant than their monomicrobial counterparts. Observed in polymicrobial biofilms of medical device infections, *Candida albicans* secretes β-1,3-glucan, protecting *S. aureus* from vancomycin by forming a dense matrix. Concurrently, *S. mutans* produces glucans that sequester fluconazole, enhancing *C. albicans* tolerance [67]. Similar synergistic effects can also be seen with *E. coli* and *Enterococcus faecalis*. *E. faecalis* secretes L-ornithine, which induces *E. coli* to increase the production of siderophores like enterobactin, salmochelin, and yersiniabactin under iron-limiting conditions [68]. These siderophores help *E. coli* to scavenge iron more efficiently, promoting growth and increasing resistance against antibiotics such as aminoglycosides or immune defenses such as lipocalin-2.

Another mechanism that enhances the pathogenicity of polymicrobial biofilms is microbial synergy, where one microbe weakens host immunity, allowing another to become more pathogenic. For instance, *S. aureus* produces alpha toxin (α-toxin), a pore-forming cytotoxin encoded by the *hla* gene, which binds to the ADAM10 receptor on host immune cell membranes [69,70]. This binding triggers the oligomerization of α-toxin monomers, forming a heptameric pore that disrupts the cell membrane, leading to cell lysis and death. The resulting disruption compromises the physical barrier, facilitating *P. aeruginosa* invasion. The lysis of immune cells, including neutrophils, macrophages, and T cells, by α-toxin diminishes the host’s immune response, further increasing susceptibility to *P. aeruginosa* pathogenicity.

Commensal-like interactions also occur in polymicrobial infections, where one member of the community creates conditions that support the survival of others in otherwise inhospitable environments. For example, in oral microbial communities, the aerobic bacterium *Neisseria subflava* consumes oxygen, thereby lowering the oxygen concentration and creating a more favorable environment for anaerobes like *Fusobacterium nucleatum*, *Veillonella dispar, Porphyromonas gingivalis*, and *Prevotella nigrescens* to survive and even proliferate under aerated conditions [71,72]. Aside from aerobic commensalism, metabolic commensalism like that of *Veillonella* species and *Streptococcus oralis* within dental plaque biofilms have also been observed, where *S. oralis* produces lactic acid through carbohydrate fermentation, which is then utilized by *Veillonella* species as an energy source [73].

Efflux pumps, membrane proteins that expel antibiotics and other toxins out of bacterial cells, are also influenced by QS and crucial in reducing intracellular antibiotic concentrations and thus conferring resistance [74–76]. These pumps, such as those in the ATP-binding cassette (ABC) and resistance-nodulation-division (RND) families, actively transport a wide range of substrates out of bacterial cells [76]. Efflux pumps structurally consist of three main components: an inner membrane transporter that binds substrates, an outer membrane channel that expels substances out of the cell, and a periplasmic adaptor protein that links the two, facilitating substrate transfer across the periplasmic space. The overexpression of efflux pumps in biofilm-forming bacteria is often associated with increased resistance to multiple antibiotics, making infections difficult to treat [76]. In *E. coli*, QS regulates cell division and also controls multidrug resistance by activating the AcrAB efflux pump via QS molecule SdiA [77]. Overproduction of SdiA increases AcrAB levels and drug resistance, conferring resistance to a broad range of antibiotics, including fluoroquinolones, chloramphenicol, and tetracyclines. Conversely, sdiA null mutants show drug hypersensitivity and reduced AcrB protein. QS and efflux pumps are not unique to *E. coli*; in *S. aureus*, QS systems like Agr regulate the expression of the NorA efflux pump, which expels fluoroquinolones [78].

Another method antimicrobial resistance may develop is through horizontal gene transfer (HGT) within biofilms, allowing bacteria to exchange genetic material, including antibiotic resistance genes such as *mecA* or *tet* [79–81]. For instance, QS can upregulate the expression of *tra* genes, which are essential for the formation of conjugative pili, or *com* genes, that are critical for competence development and DNA uptake during transformation [82]. HGT in polymicrobial biofilms enables interspecies genetic exchange, resulting in the emergence of resistance in evolutionarily distant species. This process facilitates a range of adaptations. For example, conjugative plasmids, which can induce and stimulate biofilm development, have been reported to carry the carbapenemase resistance gene (*blaOXA-23*) from *Acinetobacter johnsonii* to *E. coli* 25DN via conjugation, conferring resistance to carbapenem antibiotics [83]. HGT also facilitates the exchange of mobile genetic elements, such as the *mcr-1* gene, which encodes a phosphoethanolamine transferase enzyme that provides resistance to colistin, a last-resort antibiotic. The *mcr-1* gene is often carried on plasmids that can be transferred between different bacterial species, including *E. coli* and *Klebsiella pneumoniae* [84].

Finally, QS can regulate expression of β-lactamase genes in specific bacterial species, enhancing their resistance to certain antibiotics. In *P. aeruginosa*, the QS system upregulate the production of AmpC β-lactamase, while in *Acenetobacter baumannii*, the AbaR QS system controls the expression of the *blaPER-1* gene, which encodes the PER-1 β-lactamase. β-lactamases are enzymes that break down β-lactam antibiotics, which target bacterial cell walls [85,86]. This enzymatic activity protects both the producer cells and neighboring cells within polymicrobial biofilms. For example, when *Moraxella catarrhalis*, a producer of BRO β-lactamase, is co-cultured with *Streptococcus pneumoniae* and *Haemophilus influenzae*, the BRO β-lactamase inactivates amoxicillin, thereby increasing the survival of the otherwise susceptible bacteria [87]. Other common examples of β-lactamase-producing strains include Gram-negative *bacilli* and *S. aureus* [88]. β-lactamases producing bacteria can secrete various β-lactamases such as TEM, SHV, and CTX-M types [88,89]. TEM and SHV enzymes are typically found in *E. coli* and *K. pneumoniae*, where they confer resistance to penicillins and early-generation cephalosporins by hydrolyzing the β-lactam ring. Meanwhile, CTX-M types are more efficient against expanded-spectrum cephalosporins and has become prevalent in many *Enterobacteriaceae* species.

## Quorum sensing in Polymicrobial infections

Quorum sensing (QS) in polymicrobial infections represents a complex and dynamic interplay between different bacterial species, significantly influencing their behavior, pathogenicity, and resistance to antibiotics. In polymicrobial communities, QS-mediated interactions facilitate both cooperative and competitive behaviors [3,21,90]. This can impact the microbial community’s structure and function. Cooperation often involves sharing QS signals or modulating each other’s QS systems, as seen in cystic fibrosis (CF) lung infections where *P. aeruginosa* and *Burkholderia cepacia* complex (BCC) frequently co-infect patients, with each species being able to sense and respond to the other’s QS molecules [91–93]. Other QS interspecies cooperation include metabolic cross-feeding, where one species produces metabolites that enhance the virulence of another. For instance, *Streptococcus gordonii* generates peroxide that prompts *Aggregatibacter actinomycetemcomitans* to produce a factor H-binding protein, which protects it from complement-mediated lysis [94]. Additionally, *A. actinomycetemcomitans* can utilize the lactic acid produced by *S. gordonii* as energy, further enhancing its pathogenicity. In periodontal infections, *Fusobacterium nucleatum* and *Prevotella intermedia* generate ammonia, which raises the pH to a level suitable for *Porphyromonas gingivalis* [95]. *F. nucleatum* also increases carbon dioxide levels, which further boosts the pathogenicity of *P. gingivalis* [96].

Conversely, QS can mediate competitive interactions, such as in dental plaque biofilms, where oral bacteria like *S. mutans* and *S. gordonii* compete for resources [97]. *S. mutans* produces competence-stimulating peptides (CSPs) to promote genetic diversity, while *S. gordonii* produces hydrogen peroxide to inhibit *S. mutans* biofilms. On the other hand, the CSPs secreted by *S. mutans* can trigger the uptake of exogenous DNA, enhancing its own fitness and genetic diversity. This causes a dynamic competitive balance within the dental plaque, where the antagonistic interactions can cause homeostasis of the overall composition of the biofilm community. These interactions highlight QS’s role in not just maintaining cooperative but also competitive dynamics within polymicrobial environments.

The interactions between fungi and bacteria introduce an additional layer of complexity to microbial dynamics, manifesting as endosymbiotic, synergistic, or antagonistic relationships [21]. For example, the plant pathogen *Rhizopus microsporus* maintains an endosymbiotic association with the Gram-negative bacteria *Burkholderia rhizoxinica* and *Burkholderia endofungorum*, which are responsible for producing rhizoxin, the toxin that leads to rice seedling blight. In the context of dental plaque, fungi and bacteria often engage in synergistic interactions that facilitate biofilm development. On the other hand, certain fungi, such as those from the *Penicillium* genus, can secrete quorum-sensing inhibitors, disrupting bacterial communication and thereby diminishing the virulence of competing bacteria. Among the identified quorum-sensing molecules produced by fungi are peptides like the quorum-sensing-like peptide 1 from *Cryptococcus neoformans*, oxylipins found in *Aspergillus nidulans*, and alcohol derivatives such as tyrosol, which triggers filamentation in *C. albicans* [21].

**Figure 2.**
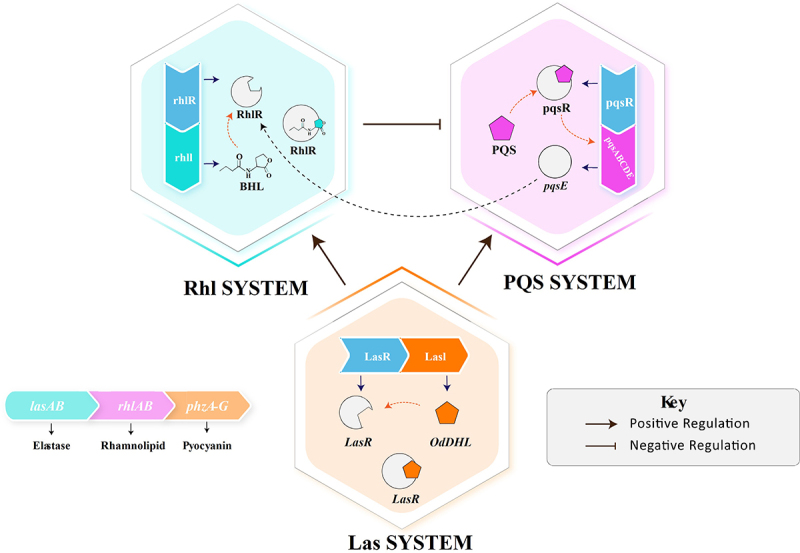
Hierarchical quorum sensing in *P. aeruginosa* Integrating Rhl, las, and PQS systems.

One of the most well-studied polymicrobial interactions involving quorum sensing occurs between *S. aureus* and *P. aeruginosa* in cystic fibrosis (CF) infections (Figure 2). Quorum sensing (QS) molecules produced by *P. aeruginosa*, such as 4-hydroxy-2-heptylquinoline-N-oxide (HQNO), inhibit the electron transport chain of *S. aureus*, leading to metabolic alterations and the formation of small colony variants (SCVs) [98]. The switch to SCVs is a hallmark of chronic infections and allows *S. aureus* to persist in the face of host immune responses and antibiotic treatment [99]. Furthermore, *P. aeruginosa*‘s QS-regulated factors, such as pyoverdine and pyochelin (two siderophores), can inhibit *S. aureus* growth and reduce its susceptibility to antibiotics [98,100]. These interactions between the two pathogens contribute to a complex dynamic where *P. aeruginosa* disrupts *S. aureus* biofilms yet simultaneously selects for phenotypes, such as the mucoid form of *S. aureus*, that are better equipped to survive in the hostile lung environment and more resistant to vanomycin [101]. Conversely, co-culture *S. aureus* can also produce exoproducts like staphylopine (StP) and staphylococcal protein A (SpA) that are detected by and affect *P. aeruginosa* biofilm formation. StP reduces *P. aeruginosa* biofilm biomass by sequestering metals like zinc, which are critical for biofilm stability [102]. In contrast, SpA can interact with *P. aeruginosa*‘s exopolysaccharide (Psl) and type IV pili (PilA) to promote bacterial aggregation and biofilm formation [103]. Importantly, these polymicrobial interactions are subject to strain-specific variations, highlighting the adaptive co-evolution of these pathogens within the CF lung environment [104].

**Figure 3.**
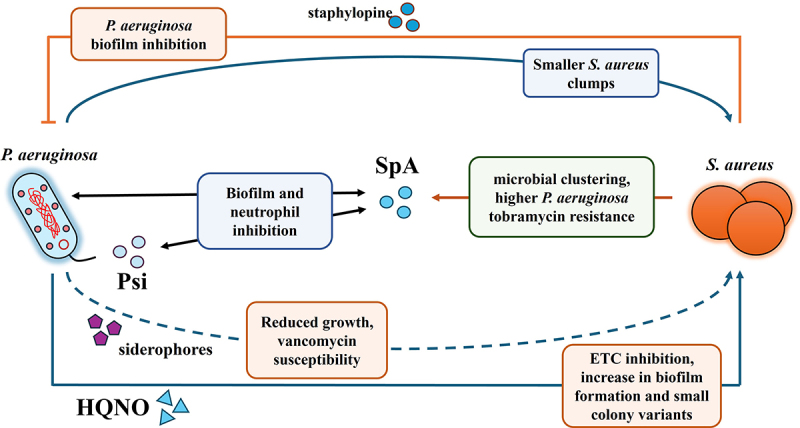
Polymicrobial interactions between *P. aeruginosa* and *S. aureus* enhancing biofilm and antibiotic resistance.

## Laboratory models

Laboratory models are essential for studying quorum sensing (QS) and its effects on bacterial behavior and antibiotic resistance. These models range from simple in vitro systems to more complex dual-species cultures that mimic polymicrobial infections. In vitro biofilm models typically involve growing bacterial cultures on various surfaces under controlled conditions, allowing researchers to observe biofilm development and QS signaling [105]. Common substrates for biofilm growth include glass, plastic, and biological materials such as mucin or collagen [105].

Static models are among the most commonly used in vitro systems due to their simplicity, cost-effectiveness, and ability to facilitate high-throughput analysis. These models operate in a closed environment where nutrients are not replenished during the experiment, which can limit the long-term viability and maturation of biofilms but provides a controlled setting to study initial biofilm formation and interactions. One frequently used in vitro static biofilm model is the microtiter plate assay. In the microtiter plate assay, bacteria are grown in microtiter plate wells, enabling easy quantification of biofilm biomass using staining techniques like crystal violet staining [106]. Through this model, researchers can manipulate environmental conditions such as iron concentration or glucose levels, or introduce QS inhibitors to study their effects.

Another widely used static model is the Calgary biofilm device (CBD) [107]. The CBD builds on the microtiter plate assay by incorporating a pegged lid, which allows biofilms to form on the pegs themselves. This design provides the flexibility to transfer the biofilms to fresh media or different environments with minimal disruption, making it particularly useful for testing the effects of antimicrobial agents and other treatments on established biofilms-associated infections. Compared to the microtiter plate, the CBD offers the advantage of intact biofilm structures during experimental manipulation as detaching the biofilm from the pegs is not necessary during transfer.

While static models like the microtiter plate assay and Calgary biofilm device are valuable for high-throughput screening and studying initial biofilm formation, they are limited by the finite nutrient supply and the absence of fluid dynamics. To overcome these limitations, dynamic models have been developed. Dynamic models, also known as open-system models, provide a continuous supply of nutrients and simulate the natural environment more accurately by introducing shear forces and fluid flow, which are essential for mimicking the conditions that many biofilms experience in vivo [105].

**Figure 4.**
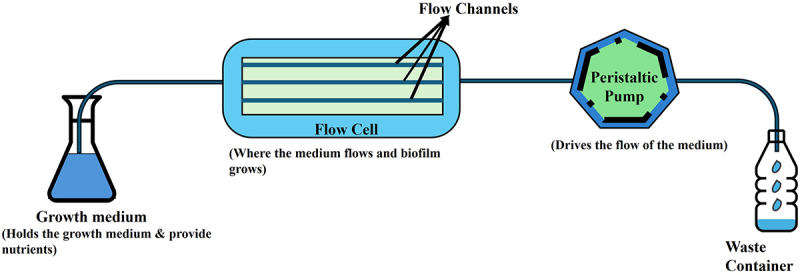
Basic flow cell system.

Flow cell systems, as shown in Figure 4, are a dynamic in vitro model that enables researchers to maintain a continuous flow of nutrients while simultaneously removing waste products [108]. This setup more accurately simulates the conditions under which biofilms form in vivo, such as those found in dental plaque or sinus infections. Flow cells are often used to study QS-regulated biofilm development in bacteria such as *P. aeruginosa* and *Staphylococcus cerevisiae* [109]. Two variations of flow cell systems are Robbins devices and drip flow reactors [110].

The Robbins device is designed to allow biofilm formation on removable plugs that are exposed to a flowing liquid [110,111]. These models are useful for studying the early stages of biofilm formation and the effects of different flow rates on biofilm development. Researchers begin by allowing planktonic cells to adhere under low-flow conditions, followed by increasing the flow rate to study biofilm maturation. This makes the Robbins device a valuable tool for investigating infections involving vascular or urinary systems such as that of infective endocarditis involving *S. aureus* or *S. mutans*. Drip flow reactors, on the other hand, utilize a low-shear fluid flow [111]. In this model, liquid drips continuously down an angled substratum, creating a gradient of nutrients and shear forces across the biofilm surface. This setup is particularly suited for studying biofilms in environments with low flow, such as in chronic wound infections or on medical devices like catheters. The uneven distribution of nutrients and exposure to air or gases at the air-liquid interface in drip flow reactors promotes biofilm heterogeneity, further allowing it to be a useful tool for exploring the effect different environmental conditions can have on biofilm formation.

In contrast, dual-species culture models are designed to study QS interactions between different bacterial species. These models are crucial for understanding how QS signals influence interspecies communication and cooperation in infections containing multiple microbes. In dual-species static biofilm models, two bacterial species are co-cultured on a substrate [110]. This allows researchers to observe interactions such as competition or cooperation. These systems have been employed to investigate how *E. coli* and *Proteus mirabilis* communicate through AI-2 signaling, influencing antibiotic resistance and biofilm formation [112]. Flow cell systems can also be adapted for dual-species cultures, closely mimicing environments such as the oral cavity or medical devices [113]. Flow cell systems have been used to study interactions between *P. aeruginosa* and *B. cepacia* complex in cystic fibrosis lung infections [114].

Despite valuable insights provided by these in vitro models, they often cannot fully replicate the complexity of an in vivo environment, where interactions with host tissues, the immune system, and microbial communities play a role in the progression of biofilm-associated infections. To address these limitations, in vivo models have been employed to study polymicrobial biofilms in the context of a living organism, offering a more complete representation of infection dynamics.

Invertebrate models, such as *Caenorhabditis elegans* (nematode), *Drosophila melanogaster* (fruit fly), and *Galleria mellonella* (greater wax moth), are increasingly used for biofilm research due to their low cost, ease of maintenance, and the ability to conduct high-throughput studies [115,116]. For example, *C. elegans* has been used to study how biofilm formation by *P. aeruginosa* affects its pathogenicity, while *G. mellonella* has been employed to explore the role of *C. albicans* and *S. aureus* in polymicrobial infections [117]. The primary disadvantage of using invertebrate models is that they may lack the complexity and relevance of mammalian systems when studying biofilm-associated infections in humans.

Mammalian models, on the other hand, provide a more physiologically relevant environment for studying polymicrobial biofilms, as they more closely mimic human disease conditions. Rodents, such as mice and rats, are the most commonly used mammalian models due to their relatively low cost, availability of genetic tools, and similarities to human anatomy and physiology. These models are frequently used to study chronic wound infections, cystic fibrosis lung infections, and catheter-associated urinary tract infections (CAUTIs). For instance, mouse wound models have been employed to investigate the synergistic interactions between *S. aureus* and *P. aeruginosa* in chronic wound infections, as well as the impact of *P. aeruginosa* and *Burkholderia cenocepacia* on lung function in cystic fibrosis patients [118,119]. The use of mammalian models allows for the examination of biofilm behavior in the presence of a functional immune system, providing insights into host-pathogen interactions.

## Strategies for disrupting quorum sensing for polymicrobial infections

Disrupting quorum sensing (QS) is emerging as a promising strategy for managing the complex challenge of polymicrobial infections, where the interaction of multiple bacterial species often complicates treatment. By interfering with QS pathways, it is possible to reduce bacterial virulence, inhibit biofilm formation, and boost the effectiveness of conventional antibiotics. Quorum sensing inhibitors (QSIs) achieve these effects by targeting the communication systems that bacteria use to coordinate harmful behaviors, making them an attractive alternative or complement to traditional antibiotics. Understanding the mechanisms through which QSIs operate, assessing their effectiveness in clinical studies, and evaluating their efficacy specifically in polymicrobial infections are important in harnessing their full therapeutic potential.

## Mechanisms of QSIs

Quorum sensing inhibitors (QSIs) employ a variety of mechanisms to disrupt bacterial communication systems, effectively reducing the virulence and biofilm-forming capabilities of pathogens. These mechanisms can be broadly categorized into inhibitors that target QS signaling molecules, targeting QS signal molecule receptors, or blocking the downstream signaling cascade after QS signal molecule-receptor binding. Understanding these mechanisms is important for developing more effective QSIs and enhancing their application.

One of the primary mechanisms QSIs may act involves targeting the signal molecules, such as autoinducers (AIs), to disrupt the QS system. This approach typically employs enzymes like AHL-lactonases and AHL-acylases, oxidoreductases, and antibodies, which degrade AIs, thereby preventing them from activating QS pathways [120]. For example, AHL-lactonases from the metallo-β-lactamase superfamily, such as the enzyme encoded by the *aiiA* gene in *Bacillus* species, can degrade AHLs in Gram-negative bacteria [121]. Similarly, antibodies can neutralize autoinducing peptides (AIPs) in Gram-positive bacteria, such as the neutralization of AIP-4 by monoclonal antibodies in *S. aureus* [122]. Additionally, oxidoreductases like the NADP-dependent reductase BpiB09 can alter AIs to reduce their binding affinity to receptors, leading to decreased biofilm formation and virulence [123]. In addition to degrading or modifying existing AIs, inhibiting the biosynthesis of QS signaling molecules provides a direct approach to disrupting the QS system. Some QSIs target AI synthases, such as TofI in *Burkholderia glumae*, where AHL analogs can bind to the active site and disrupt AHL synthesis. Similarly, diketopiperazines can inhibit CepI, an AHL synthase in *B. cenocepacia* [124]. These mechanisms of degrading, neutralizing, or inhibiting synthesis of QS signaling molecules provide a direct way to inhibit a QS system.

While targeting signal molecules represents a direct approach to disrupting quorum sensing (QS) pathways, another effective strategy involves targeting the receptors of these signaling molecules. By targeting receptors, inactivation of QS pathways can occur even in the presence of intact signaling molecules, adding an additional layer in QS disruption. The ligand-binding domains of QS receptors are often highly conserved, allowing for competitive and noncompetitive binding. For instance, flavonoids like naringenin, a plant-derived compound, compete with the native signaling molecule OdDHL for binding to the LasR receptor in *P. aeruginosa*, thereby preventing the production of factors such as pyocyanin and elastase [125]. Similarly, the anti-diabetic drug sitagliptin also interacts with LasR, and even at low concentrations, significantly reduces biofilm formation through competitive binding [126]. Furanones, another class of QSIs, can outcompete native AIs for receptor binding, effectively blocking AHL receptors across multiple bacterial species such as *P. aeruginosa, E. coli, V. cholerae*, and *S. aureus* [120]. In Gram-positive bacteria, synthetic peptide analogs have been designed to antagonize peptide-based QS systems by blocking the binding of AIPs to the AgrC receptor, such as in the Agr system of *S. aureus* [127]. In addition to competitive interactions, noncompetitive inactivation of QS receptors can also disrupt QS pathways independent of ligand activity. For instance, certain flavonoids can non-competitively bind to LasR, preventing its attachment to DNA and repressing QS-related behaviors. Moreover, certain QSIs can target multiple receptors simultaneously. For example, 3-benzene lactic acid (PLA) produced by *Lactobacillus* binds antagonistically to both the RhlR and PqsR receptors in *P. aeruginosa*, exhibiting higher affinity than the native ligands C4-HSL (BHL) and PQS [120].

A third approach in quorum sensing (QS) inhibition involves blocking the signaling cascade. This method focuses on disrupting the downstream response regulators or other essential regulatory elements within the QS system, thereby preventing the transcriptional activation of genes responsible for virulence and biofilm formation. For instance, in *S. aureus*, phosphorylation activates the response regulator AgrA, which then binds to promoter DNA, driving the expression of various genes. While savarin can bind to AgrC to prevent possible activation of AgrA, savarin can also directly target AgrA at a later stage in the QS pathway, disrupting the *agr* operon and preventing gene expression in *S. aureus* [128]. Additionally, QSIs can target other regulatory factors involved in the signaling cascade. For example, virstatin inhibits the expression of virulence factors in *V. cholerae* by repressing AnoR, a positive regulator of the LuxI-like synthase AnoI in *Acinetobacter nosocomialis* [129]. This repression decreases the synthesis of the signaling molecule *N*-(3-hydroxy-dodecanoyl)-L-homoserine lactone (OH-dDHL). Moreover, efflux pump inhibitors like PAβN reduce the extracellular accumulation of QS signaling molecules in *P. aeruginosa*, significantly downregulating the expression of QS cascade genes such as *pqsA, pqsR, lasI, lasR, rhlI*, and *rhlR* [130].

## Effectiveness of QSIs in clinical studies

There is a growing body of evidence supporting the effectiveness of quorum sensing inhibitors (QSIs) in clinical settings, building on substantial in vitro and laboratory research that demonstrates their ability to disrupt biofilm formation and reduce bacterial virulence.

One randomized placebo-controlled trial conducted by van Delden et al. evaluated the impact of azithromycin, a macrolide antibiotic with quorum sensing inhibitory properties, on patients with ventilator-associated pneumonia (VAP) caused by *P. aeruginosa* [131]. The study specifically targeted patients colonized by strains producing high levels of the quorum sensing-regulated virulence factor, rhamnolipids, which significantly increases the risk of developing VAP. The results demonstrated that while the overall reduction in VAP incidence with azithromycin treatment did not achieve statistical significance in the general cohort (4.7% in the azithromycin group versus 14.3% in the placebo group, *p* = .156), a substantial fivefold reduction in VAP incidence was observed among the high-risk subgroup of patients colonized by rhamnolipid-producing isolates (20% in the azithromycin group versus 100% in the placebo group, *p* = .048). This suggests that the efficacy of azithromycin in preventing VAP may be particularly pronounced in patients colonized by quorum-sensing proficient strains

In another clinical trial, Zhu et al. [295] investigated the safety and efficacy of fimbrolide-coated contact lenses, designed to inhibit quorum sensing, in both human volunteers and guinea pigs [132]. Fimbrolides are a class of synthetic compounds that mimic natural furanones, disrupting quorum sensing by competitively inhibiting the binding of acyl-homoserine lactones (AHLs) to LuxR-type receptors in Gram-negative bacteria. The study demonstrated that the fimbrolide-coated lenses significantly reduced bacterial adhesion across several pathogens, including *P. aeruginosa* (by 67%, *p* < .001), *S. aureus* (by 92%, *p* < .001), and *Serratia marcescens* (by 87%, *p* < .01), as well as *Acanthamoeba castellanii* trophozoites (by 70%, *p* < .01). These reductions were observed after 24 hours of lens wear in humans and during a 30-day continuous wear period in guinea pigs.

Fong et al. conducted a comprehensive study investigating the quorum sensing inhibitory (QSI) activity of itaconimides, focusing on their impact on the Las, Rhl, and PQS quorum sensing systems in *P. aeruginosa* [133]. Itaconimides were found to significantly reduce the expression of key QS-regulated virulence factors, including elastase, rhamnolipid, and pyocyanin. Specifically, the most potent itaconimide analog, 18a, exhibited IC50 values of 0.17 ± 0.04 µM for LasB-gfp, 0.61 ± 0.04 µM for RhlA-gfp, and 0.143 ± 0.13 µM for PqsA-gfp, indicating strong inhibition across these pathways. Additionally, when combined with the antibiotic tobramycin, itaconimides demonstrated a synergistic effect, leading to the eradication of *P. aeruginosa* biofilms. This was particularly evident with compound 12a, which, when used in combination with tobramycin, resulted in the complete killing of biofilm-embedded bacterial cells, a feat that tobramycin alone could not achieve. The study further confirmed that these compounds were non-cytotoxic to murine macrophages at concentrations up to 40 µM, highlighting their potential as a safe and effective anti-virulence therapeutic strategy for treating *P. aeruginosa* infections.

Quorum sensing inhibitors (QSIs) derived from natural compounds have also garnered significant attention in clinical research.

Terpenoids, a diverse class of natural compounds, have been the focus of several clinical studies exploring their quorum sensing inhibitory properties. In 2011, a randomized, multicenter parallel group trial involving 232 women evaluated the efficacy of a vaginal douche containing two monoterpenes, thymol and eugenol, in treating bacterial vaginosis (BV) across 23 gynecological centers [134]. Both thymol and eugenol are known to exhibit quorum sensing inhibitory (QSI) properties. Administered once daily for a week, the treatment led to a significant reduction in vaginal pH, itching severity, and inflammatory symptoms.

Furthermore, a clinical trial conducted by Shim et al. explored the effects of β-caryophyllene, a natural sesquiterpene, for its anti-inflammatory, antimicrobial and quorum-sensing inhibiting properties in patients infected with *Helicobacter pylori* [135]. This randomized, double-blind, placebo-controlled study involved 66 participants, with 33 patients receiving 126 mg/day of β-caryophyllene over an 8-week period. β-caryophyllene significantly reduced gastrointestinal symptoms and serum levels of the proinflammatory cytokine IL-1β (*p* = .038). This reduction in IL-1β levels suggests that β-caryophyllene’s inhibition of quorum sensing may be reducing the inflammatory response typically exacerbated by *H. pylori*’s QS-regulated virulence factors, such as urease and cytotoxins.

Flavonoids are another class of natural compounds that have been extensively studied for their quorum sensing inhibitory properties. For instance, in dental health, the antibacterial and quorum sensing inhibitory properties of baicalein, a flavone from *Scutellaria baicalensis*, has been studied. Yi et al. investigated baicalein’s effects on dentin bond durability in conjunction with ethanol-wet bonding [136]. The study treated 60 human molars with varying concentrations of baicalein in ethanol (0.01%, 0.05%, and 0.1%) and found a dose-dependent antibiofilm effect against *S. mutans*, the primary bacterium responsible for dental caries. The 0.1% baicalein group exhibited a more than 50% reduction in biofilm biomass compared to controls, with a significant decrease in bacterial metabolic activity (*p* < .05) [136].

The effects of epigallocatechin gallate (EGCG), a catechin flavonoid, were evaluated in a randomized clinical trial involving 47 children aged 5–12 years who were at high risk for dental caries. The study found that rinsing with a 4000 μg/mL EGCG solution for one minute resulted in a significant reduction in the concentrations of S. *mutans* and *lactobacilli*, key contributors to tooth decay [137].

## Efficacy of QSIs in polymicrobial infections

QSIs, whether alone or in combination, have also shown potential in being highly effective against polymicrobial infections. Pekmezovic et al. (2021) conducted a study on the anti-quorum sensing activity of essential oils extracted from citrus fruits, specifically pompia (Citrus × monstruosa) and grapefruit (Citrus × paradisi), against polymicrobial biofilms composed of *P. aeruginosa* and pathogenic fungi such as *Aspergillus fumigatus* and *Scedosporium apiospermum* [138]. The study demonstrated that these essential oils significantly inhibited the formation of both monomicrobial and polymicrobial biofilms at concentrations as low as 10 mg/L. Notably, the inhibition was particularly effective against biofilms containing *P. aeruginosa* and *A. fumigatus*, where biofilm formation was reduced by up to 70% with the grapefruit essential oil and similar reductions with pompia oil at the same concentration. The essential oils functioned by disrupting quorum sensing pathways in *P. aeruginosa*, and permeabilizing the fungal cell membranes, with the study finding that pompia oil inhibited the production of C4-HSL by 65% and 3OC12-HSL by 45%, while grapefruit oil primarily reduced long-chain AHL production by 20% [138].

Furthermore, a study by Murray et al. (2022), demonstrated the efficacy of quinazolinone (QZN) 34, a PQS quorum-sensing inhibitor, when used in combination with the aminoglycoside antibiotic tobramycin [139]. Focusing on biofilms formed by *P. aeruginosa* and *S. aureus*, the study revealed that QZN 34 at 50 µM caused a 3-log reduction in *S. aureus* viability within the biofilm in just 3 hours. When combined with tobramycin (100 µg/mL), the treatment further reduced *S. aureus* biofilm biomass by 4-fold. For *P. aeruginosa*, QZN 34 reduced biofilm biomass by 52% and decreased extracellular DNA (eDNA) production by 60%. In the mixed-species biofilm, the combined treatment of QZN 34 and tobramycin eradicated over 98% of the biofilm biomass, disrupting the biofilm matrix and significantly reducing both bacterial species [139]. This synergistic effect was attributed to QZN 34’s ability to disrupt quorum sensing in *P. aeruginosa* and perturb the membrane potential in *S. aureus*,

Saw palmetto oil has shown efficacy in inhibiting the formation of dual and three-species biofilms involving *S. aureus*, *E. coli* O157, and *C. albicans* [140]. This effect is primarily attributed to two fatty acids found in the oil, lauric acid, and myristic acid. The fatty acids exhibited potent anti-biofilm activity without inhibiting the planktonic growth of the microbes. Lauric acid and myristic acid were found to reduce biofilm formation by up to 90% at concentrations as low as 10 µg/mL in three-species biofilms. Mechanistically, these fatty acids downregulate *hla* in *S. aureus*, *fimH* and *luxRS* in *E. coli*, and *HWP1* in *C. albicans* [140,141]. Furthermore, transcriptomic analysis revealed that these fatty acids could alter the hydrophobicity of bacterial cells, thereby reducing their ability to adhere to surfaces and form biofilms. These findings suggest that lauric and myristic acid, which are sold as over-the-counter supplements, holds promise as a natural and safe anti-biofilm agent for treating polymicrobial infections.

## Future research

Despite the promising potential of QSIs demonstrated in laboratory settings, significant challenges remain in translating these findings into clinical applications. One of the primary obstacles is the complexity of polymicrobial infections in human patients, which often differ significantly from controlled laboratory conditions. The heterogeneity of microbial communities, the presence of host factors, and the influence of the immune system can all affect the efficacy of QSIs in vivo. This discrepancy raises concerns about the reproducibility of laboratory results in real-world clinical environments.

Additionally, the regulatory pathways for approving QSIs as therapeutic agents present another significant challenge. Unlike traditional antibiotics, QSIs are a novel class of therapeutics with mechanisms that are not yet fully understood. The scalability of QSI production for clinical use is also a concern, as the manufacturing processes developed for laboratory-scale studies may need to be significantly adapted to meet clinical-grade standards and the demands of widespread use. Addressing these challenges will be critical to realizing the therapeutic potential of QSIs in treating polymicrobial infections.

Another major challenge is the pharmacokinetic and pharmacodynamic profiles of QSIs, which remain poorly understood in clinical contexts [2,142]. The effective concentration of QSIs in human tissues, their stability, bioavailability, and potential off-target effects need thorough investigation. Optimal dosing regimens and delivery methods must be established to maximize the therapeutic benefits of QSIs while minimizing potential side effects.

While QSIs target communication pathways rather than directly killing bacteria, which theoretically reduces the selection pressure for resistance, there is still a risk that bacteria could evolve mechanisms to bypass QSI effects. Therefore, it is essential to monitor the emergence of resistance and develop strategies to mitigate this risk. Furthermore, the specificity of QSIs is an important consideration. Certain broad-spectrum QSIs may disrupt beneficial microbial communities, leading to unintended consequences [143]. Therefore, the development of species-specific QSIs that target pathogenic bacteria without affecting commensal microbes is a key research focus.

## Conclusion

Advancing the understanding of quorum sensing (QS) and its role in antibiotic resistance offers several promising avenues for future research and therapeutic development. Enhancing the efficacy and specificity of quorum sensing inhibitors (QSIs) for polymicrobial infections remains a key research focus, with sophisticated laboratory techniques facilitating the identification of unique QS pathways and signal molecules in pathogenic bacteria. Understanding these mechanisms will facilitate the development of more specific QSIs and, when integrated with genomic and metagenomic data, will further aid in identifying novel biosynthetic pathways. The synergistic potential of combining QSIs with traditional antibiotics holds promise, requiring future research to optimize combination therapy protocols, including dosing regimens and delivery methods, to maximize therapeutic outcomes while minimizing side effects. Exploring new sources of QSIs, such as natural products derived from plants, marine organisms, and microorganisms, is another promising direction, with the screening of natural product libraries potentially leading to the discovery of novel compounds with unique modes of action. Synthetic biology approaches can further enhance these efforts by engineering microorganisms capable of producing QSIs with improved properties. In conclusion, the future of QS research and its applications in managing antibiotic resistance in polymicrobial infections is promising. Continued research will be essential to realize the full potential of QS-based therapies and to overcome the global challenge of antibiotic resistance.

## Data Availability

As new data was not generated or analyzed in this study, data sharing is not applicable.
